# Genome sequences of 10 new carnation mottle virus variants

**DOI:** 10.1128/MRA.00189-23

**Published:** 2023-08-09

**Authors:** Timo M. Breit, Wim de Leeuw, Marina van Olst, Wim A. Ensink, Selina van Leeuwen, Martijs J. Jonker, Rob J. Dekker

**Affiliations:** 1 RNA Biology & Applied Bioinformatics research group, Faculty of Science, Swammerdam Institute for Life Sciences, University of Amsterdam, Amsterdam, the Netherlands; Katholieke Universiteit Leuven, Leuven, Belgium

**Keywords:** plant, RNA virus, CarMV, variant

## Abstract

Here, we report the genome sequences of 10 Carnation mottle virus variants. Six variants originated from a single proprietary carnation cultivar, and four were derived from four different proprietary cultivars. All variants showed nucleotide differences, but the last four did not show any variation at the amino acid level.

## ANNOUNCEMENT

*Carnation mottle virus* (CarMV, genus: *Alphacarmovirus*, family: *Tombusviridae*) is a single-stranded positive-sense RNA virus with four open reading frames (ORFs) (1). Infection of the major host for CarMV, *Dianthus caryophyllus* (carnation), can result in mild mottling in young leaves but typically only faint chlorosis in mature leaves. Besides the CarMV genome reference sequence NC_001265.2, just 11 complete variant genome sequences are present in Genbank (2, 3).

To gain deeper understanding of the CarMV genetic variation, we conducted small-RNA sequencing (sRNA-seq) on 10 asymptomatic carnation plants from various undisclosed commercial cultivars sourced from an anonymous plant-breeding company in the Netherlands. In carnation experiment 1 (CE1), six out of eight plants belonging to the same confidential cultivar were found positive for CarMV enzyme-linked immunosorbent assay (ELISA). In carnation experiment 2 (CE2), four distinct confidential cultivars that tested positive for CarMV ELISA were used. All samples were collected in February 2020 from a greenhouse complex situated in the southern region of the North-Holland province in the Netherlands. Genomes sequences of possible CarMV variants were determined by examining the siRNA response of the plants (4).

The ELISA assay was performed using the Prime Diagnostics (Wageningen, the Netherlands) CarMV Kit (https://shop.wur.nl/primediagnostics/carnation-mottle-virus-carmv.html) following the protocol provided by the manufacturer. Small-RNA was isolated by grinding a flash-frozen ±1 cm^2^ leaf fragment to fine powder using mortar and pestle, dissolving the powder in QIAzol Lysis Reagent (Qiagen) and enriching small-RNA using the miRNeasy Mini Kit (Qiagen). Barcoded small-RNAseq libraries were generated using Ion Total RNA-Seq Kit v2 and Ion Xpress RNA-Seq Bar Coding Kit (Thermo Fisher Scientific). Sequencing (150 bases) with an Ion Proton System using Ion PI-v3 chips and a Ion Chef System using Ion PI-Hi-Q-Chef Kit (Thermo Fisher Scientific) CE1 and CE2 yielded on average 5 (CE1) and 31 (CE2) million reads per sample.

The CarMV variant genomes were constructed using NC_001265.2 as a reference. First, reads were trimmed using trimmomatic v0.39 (5) [parameters: LEADING:3;TRAILING:3;SLIDINGWINDOW:4:15;MINLEN:19] and mapped to the reference using bowtie2-v2.4.1 (6). Next, Freebayes-v1.3.2 (7) was used to extract variations which were incorporated in the reference using gatk’s FastaAlternateReferenceMaker-v4.2.5.0 (8) for an updated sequence. This process was repeated until no new variations were detected. Default parameters were used except where otherwise noted.

**Fig 1:**
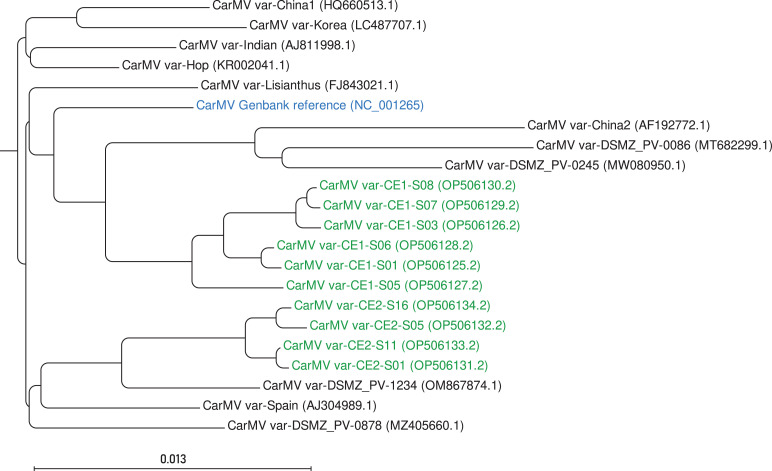
Phylogenetic analysis of CarMV variants. The study included 10 CarMV variants obtained from the current investigation (represented in green), along with 11 CarMV variants sourced from GenBank (represented in black). The CarMV reference genome sequence NC_001265 is shown in blue. The phylogenetic tree was constructed using CLC Genomics Workbench v21.0.5 software. The tree was generated by initially aligning the genome sequences of the CarMV variants, utilizing the fol­lowing gap cost settings: open 10, extension 2, and end gap “As any other.” Subsequently, the neighbor joining method was employed to construct the tree, and Kimura’s two-parameter model (K80) was utilized for estimating genetic distances. To assess the robustness of the tree, bootstrapping was performed with 100 replicates.

We determined the genome sequence of the CarMV variant in each CarMV-positive plant with a 100% coverage breadth for each sample. Compared to the CarMV reference genome (1) (NC_001265.2), mutations were observed, ranging from 108 (2.7 %) to 141 (3.5%) nucleotides (Table 1). These differences are alike those between known Genbank CarMV variants (78–187 mismatch nucleotides). In a genetic relation analysis, the CarMV variants from our two experiments cluster together (Fig. 1). Additionally, CE1 and CE2 CarMV variants exhibit a closer genetic relationship to Asian and European variants, respectively.

**TABLE 1:** DNA and protein sequence mismatches of CarMV variants compared to the reference genome NC_001265.2. Abbreviations: SRA, sequence read archive; nt, nucleotides; aDOC, average depth of coverage; aa, amino acids; RdRp, RNA-dependent RNA polymerase; RAP, replicase-associated protein; MP2, movement protein 2; MP1, movement protein 1; CP, capsid protein; ORF, open reading frame.

		Samples in carnation experiment 1 (CE1)								Samples in CE2			
		S01	S02	S03	S04	S05	S06	S07	S08	S01	S05	S11	S16
Genbank accession		OP506125		OP506126		OP506127	OP506128	OP506129	OP506130	OP506131	OP506132	OP506133	OP506134
SRA accession		SRR2175 1399	SRR2175 1398	SRR2175 1407	SRR2175 1406	SRR2175 1405	SRR2175 1404	SRR2175 1403	SRR217 51402	SRR2175 1401	SRR2175 1400	SRR2175 1397	SRR2175 1396
CarMV ELISA result		+	−	+	−	+	+	+	+	+	+	+	+
Total reads (10^6^)		6.9	4.2	5.0	4.7	5.2	5.5	5.6	5.0	27.9	26.9	28.9	40.8
CarMV reads (10^6^)		1.5		1.2		1.8	1.5	1.5	1.0	13.0	13.1	14.8	19.8
**Genome**													
Size (nt)		4,003		4,002		4,003	4,003	4,002	4,003	4,003	4,003	4,003	4,003
GC content (%)		49.1		49.1		49.2	49.1	48.8	48.8	49.0	48.9	49.0	48.8
Differences to NC_001265 (nt)		112		120		116	108	121	119	133	141	130	138
aDOC		7,700		6,100		9,500	7,900	7,900	5,500	68,300	68,900	77,400	104,000
**Protein name**	**Size (aa)**												
ORF1 RdRp	*245*	3		2		3	3	4	2	6	6	6	6
ORF1 RAP	*430*	11		8		11	10	10	8	5	5	5	5
ORF2 MP2	*84*	0		0		0	0	0	0	1	1	1	1
ORF3 MP1	*61*	1		1		1	1	1	1	2	2	2	2
ORF4 CP	*348*	6		8		8	6	6	7	4	4	4	4
All ORFs	*1,168*	21		19		23	20	21	18	18	18	18	18

The protein differences among all new CarMV variants ranged from 19 (1.6%) to 23 (2.0%) amino acids. Although there are nucleotide differences between the four CE2 CarMV variants, they are identical at the protein level, whereas the six CE1 CarMV variants are different at the genome and protein level.

## Data Availability

The raw sequence reads have been deposited in the NCBI Sequence Read Archive under BioProject accession number PRJNA885393 (Table 1). CarMV variant sequences have been deposited under GenBank accession numbers OP506125, OP506126, OP506127, OP506128, OP506129, OP506130, OP506131, OP506132, OP506133, and OP506134.
